# Approach to Management of Intravascular Missile Emboli: Review of the Literature and Case Report

**DOI:** 10.5811/westjem.2015.5.25553

**Published:** 2015-07-10

**Authors:** Kevin Lu, Sanjay Gandhi, Moqueet A. Qureshi, Andrew S. Wright, Narongrit Kantathut, Thomas P. Noeller

**Affiliations:** * Case Western Reserve University School of Medicine, Cleveland, Ohio; † MetroHealth Medical Center, Heart and Vascular Center, Case Western Reserve University School of Medicine, Cleveland, Ohio; ‡ Cleveland Clinic Foundation, Department of Surgery, Cleveland, Ohio; § MetroHealth Medical Center, Department of Emergency Medicine, Cleveland, Ohio; ¶ Cleveland Clinic Foundation, Thoracic and Cardiovascular Surgery, Cleveland, Ohio

## INTRODUCTION

Missile embolization is regarded as a rare phenomenon in the world of penetrating trauma. While figures in the world of civilian trauma do not exist, there is reason to believe that missile emboli are frequent enough to warrant the attention of any medical decision maker who cares for trauma patients. The current literature offers a variety of cases, but consolidated commentaries on management are infrequent. While a diagnostic and management plan may be pieced together with literature review, the situation in the setting of an unfolding trauma scenario often demands a more efficient approach. In this article, the authors offer a case report, as well as a review of diagnostic evaluation and management of missile emboli with support from the literature. While definitive recommendations cannot be made based on current medical and surgical understanding of missile emboli, we summarize this article by offering a likely model of managing missile emboli by anatomical location.

## CASE REPORT

A 24-year-old male with no significant prior medical history was brought to the emergency department (ED) after sustaining nine gunshot wounds (GSWs), inflicted by two assailants wielding handguns from 4–6 meters away.

Upon arrival to the ED, the patient was alert and oriented. Breath sounds were symmetric and clear, and pulse oximetry was 100%. Central and peripheral pulses were strong and symmetric. One GSW was sustained to the left buttock posteriorly. Another penetrated the right lower quadrant of the abdomen, but the rest of the abdomen was otherwise soft, non-tender, and non-distended. No deformity or bullet wound was noted in the neck, thoracic, axillary, or upper abdominal areas. Exposure of the extremities revealed GSWs to the right thigh and left arm.

**Figure 1:**
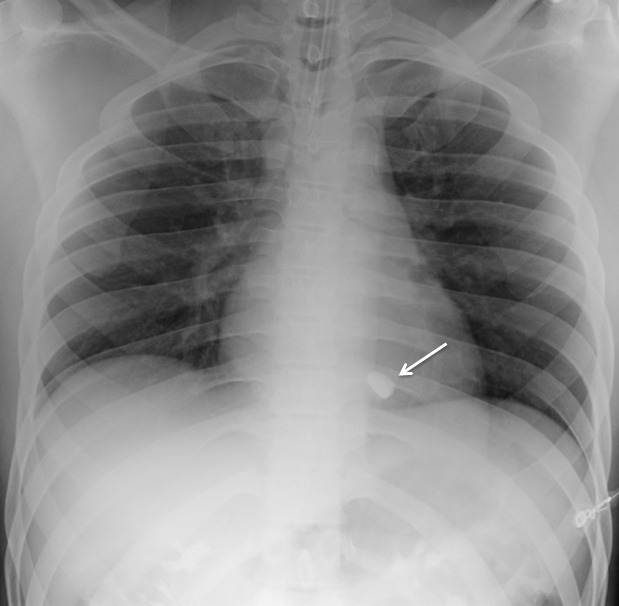
Chest radiograph showing blurred foreign body within the cardiac silhouette (arrow).

During the log roll examination of the back, SpO2 dropped to 80–90% while the patient breathed spontaneously on nasal cannula with oxygen at 2 liters per minute. The patient was positioned supine and the nasal cannula was replaced with 15 liters per minute of oxygen delivered via non-rebreather mask. After 2 minutes, his SpO2 corrected to 100% with resolution of his shortness of breath. The patient remained hemodynamically stable and the quality of the pulse oximetry waveform signal was confirmed throughout the episode. Bedside eFAST (Extended Focused Assessment with Sonography for Trauma) was negative for pneumothorax, pericardial and intra-abdominal fluid. Chest radiograph demonstrated a radiopaque foreign body measuring approximately 9×19mm, overlying the cardiac silhouette (Figure 1). The patient denied ever being shot in the past, and a preliminary concern for a bullet embolus was raised.

**Figure 2:**
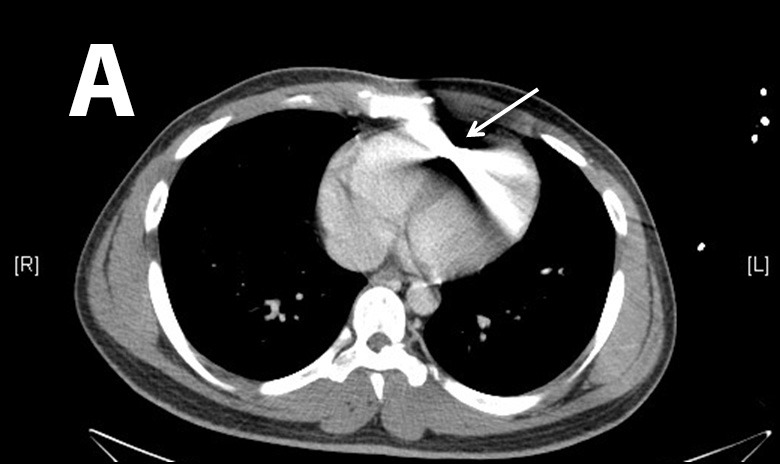

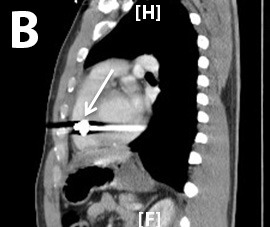
Chest computed tomograph showing bullet (arrow) in the right ventricle, both transverse (Figure 2a) and sagittal (Figure 2b) views showing significant glare.

Computed tomograph (CT) of the chest confirmed a bullet in the right ventricle (Figure 2). CT of the abdomen and pelvis along with cystogram revealed a moderate amount of acute pelvic hemorrhage with evidence of right common iliac vein injury.

**Figure 3:**
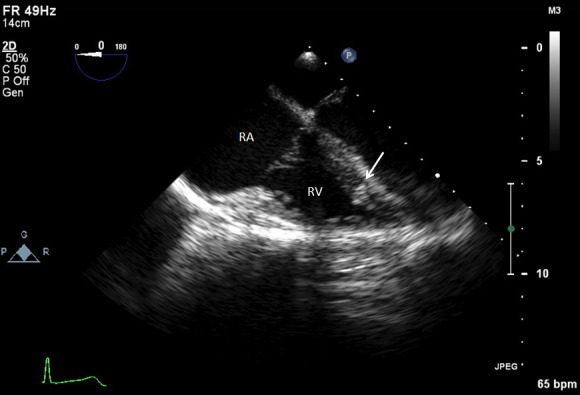

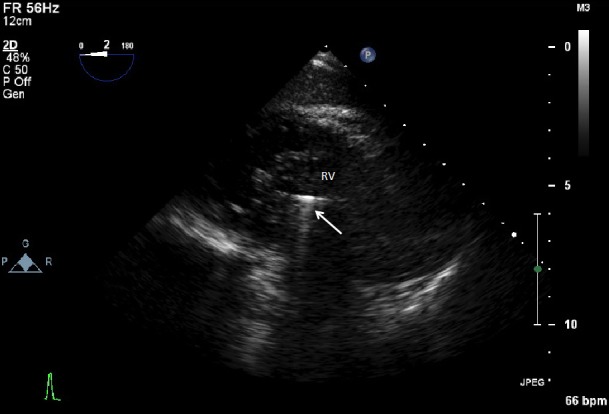
Transesophageal echocardiogram views of right ventricular (RV) apical view (Figure 3a [top]; arrow as bullet tip in RV trabeculae) and RV transverse view (Figure 3b [bottom]; arrow as bullet demonstrating significant comet tail artifact).

**Figure 4:**
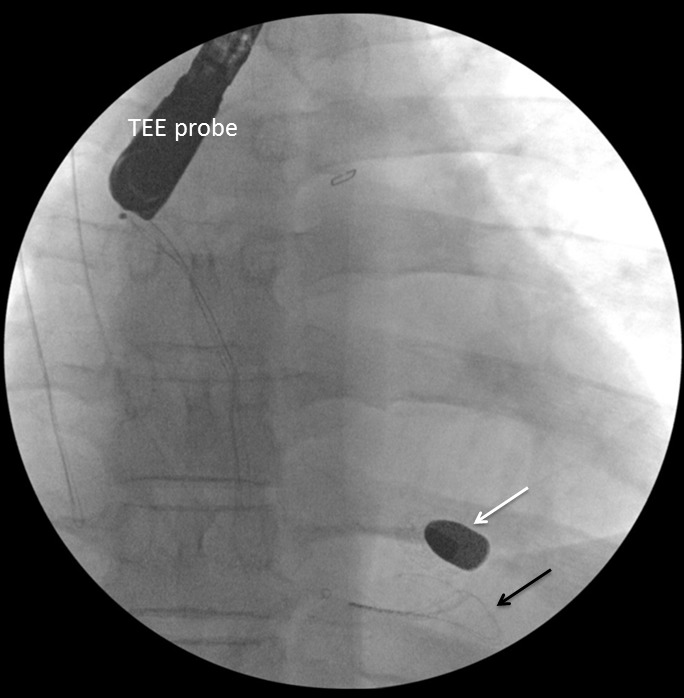
Fluoroscopy intraoperatively showing snare (black arrow) next to bullet (white arrow) and transesophageal echocardiogram probe.

Cardiothoracic surgery and cardiology were then consulted for removal of the bullet. Intraoperative transesophageal echocardiogram (TEE) was performed to confirm the location of the bullet within the right ventricle, adjacent to the ventricular septum (Figure 3 and attached .mp4 video at 0.5× speed [Supplemental Digital Content]^SDC-1^). The right ventricular transverse view on TEE showed a comet tail artifact, commonly seen with metal foreign bodies (Figure 3b). TEE further revealed normal right ventricular function, normal left ventricular function, no pericardial effusion, and no valvular abnormalities. It was uncertain after TEE whether the bullet was free floating within the ventricle at the time. Given the lack of direct damage to cardiac structures, an attempt at endovascular retrieval was made. Right internal jugular venous access was obtained by surgical cutdown, and an 11 French sheath was placed under direct visualization through the right internal jugular vein. With guided fluoroscopy, multiple attempts were made to retrieve the bullet with both Amplatz GooseNeck® (Coviden, Plymouth, MN) and ensnare devices (Figure 4). The cardiothoracic surgeons then performed a median sternotomy, and approached the right ventricle via right atriotomy with cardiopulmonary bypass and cardioplegic arrest. Exploration of the right ventricle through the tricuspid valve revealed the bullet was embedded within the right ventricular trabeculations. Extraction of the bullet required minor dissection of some trabeculations with Metzenbaum scissors. The intact bullet was discovered to be a minimally deformed .38 caliber pistol round, specifically 9×19mm Parabellum full metal jacket. The atriotomy and sternotomy were closed with no complication. The patient was transferred to intensive care in stable condition, and extubated later that day.

On POD #6, the patient developed a fever. A CT chest/abdomen/pelvis showed a large pericardial effusion, and moderate right pleural effusion with right lower lobe collapse. Echocardiogram demonstrated low-normal left ventricular ejection fraction at 50%, and furthermore confirmed the large pericardial effusion without any tamponade criteria. Clinically, the patient experienced intermittent drops in pulse oximetry and concurrent shortness of breath, but remained hemodynamically stable. Pericardiocentesis removed 650mL of hemorrhagic effusion immediately, and a drain left in place evacuated 320mL over the next three days. Follow-up echocardiogram showed a very small pericardial effusion. The 340mL of serosanginous fluid was then drained from the right pleural effusion, with no residual fluid on repeat ultrasound. There were no further acute events, and the patient was determined to be stable for transfer to the comprehensive rehabilitation center on POD #15.

## CASE DISCUSSION

This patient experienced multiple GSW to the lower extremities and pelvis. After an initially unexplained oxygen desaturation, a chest radiograph revealed a likely bullet overlying the cardiac silhouette. After the complete evaluation in the trauma bay yielded no evidence of direct thoracic penetration and negative history of prior GSWs, a suspicion was raised for bullet embolus. After CT confirmed this diagnosis, the patient underwent successful operative removal. After a complicated post-op course, the patient was transferred for intensive rehabilitation.

Our patient was shot with a .38 caliber low-velocity full metal jacket round that minimally deformed. The bullet size was the second largest recorded, with only a handful of reported .38 caliber pistol rounds of similar grain weights becoming intravascular emboli. There was only one reported instance of a .40 caliber pistol embolus, the largest bullet embolus in current literature.^1^ Evidence of injury to the right common iliac vein in our patient with no other accompanying vessel injury on imaging suggested the bullet embolized from there. Given the episode of hypoxia during the log roll, it is possible that the bullet embolized to the right ventricle at that time. Any tissue clot or air that concurrently embolized to the pulmonary vasculature likely resolved by the time CT of the chest was performed.

## DISCUSSION

Intravascular and intracardiac missile emboli, including bullets, pellets, or shrapnel secondary to mortars, grenades, and mines, are considered rare.^2^ A review of 7,500 Vietnam War missile injuries yielded 22 cases (0.3%) of missile emboli, most of which came from explosive devices.^3^ A more recent report from combined operations in Afghanistan and Iraq of 346 soldiers with vessel injury found missile emboli in 1.1%.^4^ There are no figures that accurately depict incidence in the civilian population, and the disproportionate high velocity firearm and blast injuries in the military make it difficult to extrapolate to non-military settings.

The primary factors that determine the probability of missile embolization are vessel proximity, kinetic energy, and projectile size. The kinetic energy of the projectile must be such that it enters but does not traverse the vulnerable vessel. The diameter of the object must be smaller than the intraluminal diameter of the vessel. Small, low-velocity projectiles common in civilian trauma, such as shotgun pellets, .22 caliber bullets, and air gun pellets, represent the majority of intravascular emboli.^1^,^5^ Despite a lack of reliable data confirming actual incidence, the risk of projectile embolization in the civilian realm may be more likely than in the military arena for the following reasons: 1) Low-velocity handgun and shotgun injuries are more common than wartime high velocity rifle injuries, 2) Even at several hundred meters, military assault rifle rounds are nearly twice as fast as pistol rounds at muzzle velocity,^6^ 3) Civilian rounds are typically of smaller overall size, 4) High velocity explosion injuries, uncommon in the Westernized civilian realm, comprise a large proportion of historical and modern military injuries, 5) Civilian ammunition is not restricted by the Hague Convention of 1899, meaning that bullets that fragment into smaller pieces are more common than full metal jacket military rounds.^7^ Ultimately, definitive conclusions about civilian prevalence cannot be made, but there exists the important notion that missile emboli are likely frequent enough that any practitioner in a high-volume civilian trauma center should be aware of it.

## DIAGNOSTIC EVALUATION

**Figure 5:**
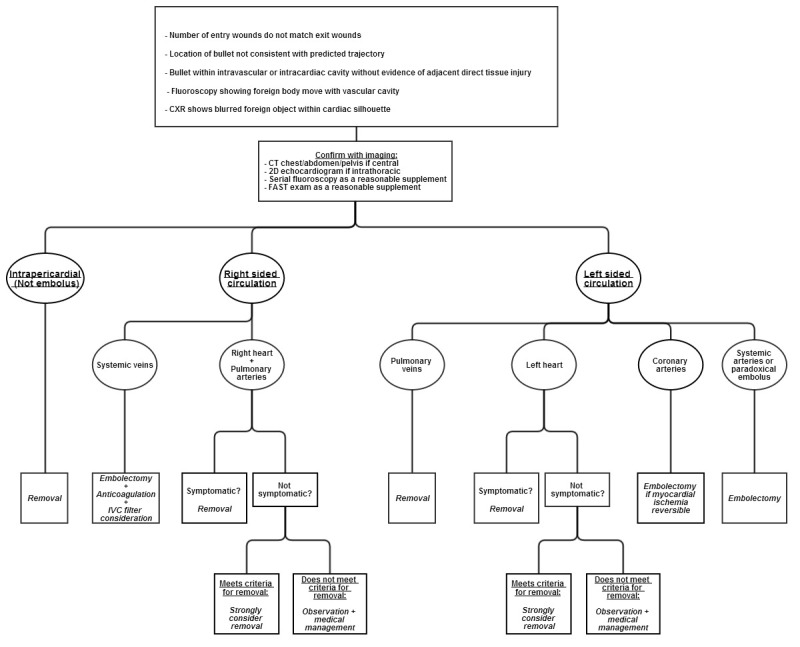
A model of missile embolus management by anatomical location based on review of the literature and authors’ own experiences. *CT*, computed tomography; *CXY*, chest x-ray; *IVC,* inferior vena cava*; FAST*, focused assessment with sonography for trauma

Suspicion of intravascular missile embolus usually begins with current presentation or history of penetrating missile trauma, alongside evidence with imaging. Other clinical factors include incongruent number of entry and exit wounds, unexpected missile trajectory, and absence of direct injury to tissue adjacent to the lingering location of the missile (Figure 5).^8^ While these factors led to the suspicion for bullet embolus in our patient upon first presentation, there have been many reports of delayed discoveries. One case described a largely uncomplicated bullet allowed to remain adjacent to the patient’s pelvic vasculature, that later was found to embolize to the right middle lobe pulmonary artery two weeks later.^9^ Another reported a 68-year-old World War II veteran who had an incidental finding of a bullet lodged in his right ventricle found upon chest radiograph imaging originally intended to visualize pacemaker placement.^10^

To determine appropriate treatment strategies, the diagnostic workup must include an accurate evaluation of size and location of the missile embolus. For intracardiac missile emboli, the first level of evidence may be a chest radiograph, which frequently shows a blurred foreign body superimposed on the cardiac silhouette.^11^ CT chest/abdomen/pelvis may help determine missile trajectory and damage to surrounding cardiac structures, but metal commonly causes scatter, making it difficult to ascertain the exact location of the foreign body.^12^ Expedient management of intracardiac emboli depends on determining whether the missile is freely mobile within a chamber, within the myocardium, within the pericardium, or nearby important cardiac structures. One fatal case was described in which specific localization was not performed prior to cardiopulmonary bypass. The authors stated that the use of 2D echocardiogram intraoperatively may have led to finding the bullet in the left atrium before it migrated in a retrograde fashion to the right pulmonary vein.^13^,^14^ TEE intraoperatively is the modality of choice for confirming intracardiac missile emboli, with TEE preferred to transthoracic echocardiogram since TEE helps better visualize the level of myocardial damage.^15^ In addition, echocardiogram sometimes demonstrates how deeply embedded the foreign body is, further affecting medical decision making. Determination of whether the intracardiac missile is left sided or right sided dictates the technical surgical approach. Clinicians must also exclude that the missile may be resting within the pericardium, since all intrapericardial missiles should be managed with surgical retrieval and antibiotics to reduce risk for pericarditis and pericardial effusions.^16^–^18^ Surgical intervention for intrapericardial missiles differs as well, since subxiphoid pericardial window is preferred to median sternotomy.^12^ Missiles within the pericardial sac are best differentiated from intracavitary missiles with serial fluoroscopy.^15^ Other than the pericardial sac, it should also be noted that missile embolization may occur within any luminal tract or potential space in the body, and that a migrating abdominal foreign body on repeat imaging may indicate an embolus within the gastrointestinal tract.^19^ Lastly, as in the case with our patient, an eFAST upon initial evaluation is a reasonable supplement to look for thoracic injuries.

Intravascular missile emboli are generally classified as arterial or venous. Currently, reports conflict regarding which type of embolus represents the majority of cases. Arterial bullet emboli are traditionally cited as more common, with figures up to 75% as arterial.^1^,^20^ Another report with over 200 compiled cases found missile emboli to be 46% arterial, 52% venous, and the rest paradoxical, which occurs when there is direct communication between the right and left heart such as a patent foramen ovale.^21^,^22^

## MANAGEMENT

Recommendations have varied widely on management of bullet emboli. Case reports numbering only in the hundreds have yielded inherently weak recommendations based primarily on anecdotal evidence. Though the authors of this article hope to present information that helps future clinicians make an informed decision, we recognize that we present only a model of an approach and not a series of guidelines.

Historically, the policy of United States armed forces in World War II was to remove intracavitary missiles from the heart, but no attempt at retrieval was to be made in patients who already stabilized after intracardiac implantation of the missile.^23^ This practice was derived from an understanding that mortality from foreign bodies in the heart was approximately 20%, but death from surgical intervention was also 20% during that time period.^24^ Dramatic surgical advancements have been made since then, with median sternotomy largely replacing thoracotomy whenever possible. There have also been many successful reports of minimally invasive endovascular retrievals of emboli since 1980, even with a case series of four from one institution that were all managed successfully with endovascular approaches.^8^,^25^

Controversy remains regarding management of intracardiac missile emboli. The rationale for leaving retained cardiac missiles comes from Fritz et al., who implanted small metal objects in dog hearts which all encapsulated the metal in fibrous tissue with minimal complication by eight weeks.^26^ Categorization of intracardiac missile emboli to left-sided versus right-sided site is traditionally deemed important for surgical management (Figure 5). All right-sided emboli have the potential to embolize to the pulmonary arteries, and all left-sided emboli may further occlude distal arterial sites. Most dangerous are the partially embedded or freely mobile intracavitary missiles. Emboli fully embedded within the myocardium are presumed to be at substantially lower risk for further embolization.^2^ To determine the combined immediate and long-term symptomatic rates of left-sided versus right-sided intracardiac emboli, we examined a combination of two articles with databases between 1940–1988 and 1990–2009. Symbas et al. reviewed 127 cases of intracardiac missiles during 1940–1988, that could be differentiated into left versus right sided and whether they were symptomatic or not.^2^ Symptomatic patients included those who either had symptoms at initial presentation or significantly after penetrating trauma injury. Our review of Symbas’ database excluded cases that either did not localize missiles, did not report whether there were symptoms or not, or reported missiles in the coronary arteries or pericardium. The addition of Lundy et al.’s data during 1990–2009 provided a total of 151 cases that reported location and symptomatic rates.^27^ Using these two databases, we determined that 18 out of 90 (20.0%) right-heart missile emboli were symptomatic or eventually symptomatic, and 17 out of 61 (27.9%) left-heart missile emboli were symptomatic or eventually symptomatic. This difference was shown to not be statistically significant even at a two-tailed confidence interval of 80% (Z-score=1.1, two-tailed probability=0.271). Interestingly, Symbas et al. and Lundy et al. at times implied that right-sided cardiac missile emboli were safe to manage conservatively, with exception of missiles freely mobile within the cardiac chamber or those that passed through contaminated viscera. However, combination of both databases revealed that right-sided and left-sided cardiac missile emboli had similar complication rates. Thus, the erroneous dismissal of a right-heart embolus as largely benign and pursuance of medical management alone may cause the clinician to inadvertently incur the same amount of risk as left-sided cardiac missile emboli. Depending on the clinician and patient, 20–30% symptomatic rates with conservative management alone may be unacceptable especially given the unpredictable nature of symptoms arising in intracardiac emboli well after immediate presentation.

There is little controversy, however, over whether arterial missiles within the vasculature require removal (Figure 5). Arterial bullets are symptomatic in 80% of cases, but venous missile emboli are symptomatic in only one third of cases.^28^ Most authors agree systemic arterial missile emboli should be removed to prevent distal ischemia, but debate persists in the literature over how venous emboli should be handled. Medical management alone of systemic venous emboli has been successful in many reports, and several authors conclude that venous emboli may be left alone if asymptomatic.^4^,^29^ Other authors have maintained that mandatory removal of venous emboli is necessary because morbidity of retained missiles is significant at 25%.^30^ Many clinicians may also find symptomatic rates at one third of cases to be unacceptable, as most patients are not immediately symptomatic, and it may be difficult to predict which venous emboli will be complicated in the future. Finally, endovascular snaring or venotomy in the periphery is preferable and much simpler in comparison to working centrally within the lungs or heart after the missile embolize further. Review of 120 cases of venous missile emboli between 1900 and 1990 showed that 83% eventually travelled to the right heart or pulmonary artery, and 4% remained in the peripheral venous system.^31^ Given the significant possibility of complications with conservative management, removal of missiles within the systemic venous circulation whether currently symptomatic or not must be placed in the context of each case (Figure 5). Proper medical management with anticoagulation for 12 months duration barring presence of significant hemorrhage, and inferior vena cava filter should also be discussed by the management team.^4^,^31^

There is a significant amount of literature about pulmonary arterial embolizations. Thirty-two cases of pulmonary artery bullet emboli were observed without complication in one article.^32^ Pulmonary artery embolization that already occurred upon discovery is frequently asymptomatic.^9^ Asymptomatic missile embolizations already resting in the pulmonary arteries may be conservatively managed since the risk following thoracotomy is greater than observing an asymptomatic patient.^8^ However, missile emboli upstream of the pulmonary arterial system must be scrutinized closely. With approximately 26% of systemic venous emboli ultimately settling in a pulmonary artery, removal of a missile embolus regardless of final resting location upstream of the pulmonary arteries must be strongly considered (Figure 5).^22^ Possible complications of retained pulmonary arterial missiles include abscess formation, infarction, erosion, or fatal exsanguination.^4^,^22^

Examples of pulmonary venous missile embolization remain sparse in the literature. As mentioned earlier, Schulman et al. reported one case in which a right pulmonary vein embolus caused fatal outflow obstruction between the right lung and left heart.^13^ While most emboli to the left heart presumably originate from the pulmonary veins, and some left-heart missiles may be managed conservatively, missile emboli retained in the pulmonary veins are dangerous. Though future case reports may give us better understanding of this phenomenon, as it stands now, all missile emboli in the pulmonary veins likely require removal (Figure 5).

The remaining types of missile embolization are rare, but whether or not to surgically intervene is less controversial. Coronary artery embolectomy should be performed if myocardial ischemia is reversible (Figure 5).^33^ Attempt should always be made to remove cerebral intravascular foreign body emboli, as mortality may be as high as 25–33% (Figure 5).^2^,^34^ Depending on the size of the missile fragment, a microsnare may be used to remove intracerebral emboli.^35^

## SPECIAL CONSIDERATIONS

Most authors recommend removal of missile emboli if the patient is symptomatic. Consequences of retained emboli include physiologic disturbances as well as psychiatric complications. Distal ischemia, thrombosis, and further embolism are the main complications of arterial missile embolus. Complications of venous missile emboli include pulmonary artery embolism, cardiac valve dysfunction, endocarditis, abscess formation, sepsis, thrombosis, dysrhythmias, intraventricular communications, conduction defects, tissue erosion, hemorrhage, cardiac ischemia from erosion into coronary vessels, and thrombophlebitis.^8^,^36^ Psychiatric consequences range from severe anxiety to cardiac neurosis, a debilitating psychological disturbance in which patients fear movement that may result in a dislodgment of the bullet from its current location.^1^,^2^,^34^,^37^

Regardless of presenting symptoms, there are certain characteristics of the mechanism of injury and the foreign body itself that should warrant serious consideration for removal for fear of future complication. Logically, minimally invasive embolectomies should also be performed if successful retrieval is highly probable based on localization. For pediatric populations, most missile emboli in the West are less traumatic, small-caliber pellet injuries requiring observation alone, but one international report on pediatric wartime injuries demonstrated a 9.5% mortality rate among 21 patients with missile emboli.^5^,^34^ In terms of defining large missile emboli, 5mm is a commonly accepted cutoff that has been adopted but it is arbitrary.^3^,^9^,^27^,^36^,^38^ Thus, we propose a more specific definition of large missiles with combined dimensions of greater than 5mm in diameter and 10mm in length. For reference, this includes all commonplace .22 caliber bullets and up, but do not include typical air gun pellets and shotgun fragments. Other characteristics prompting surgical intervention include likely embolization to distal sites, damage to adjacent tissue, and passage of missile through contaminated sites such as unclean objects external to the victim as well as abdominal viscera.^39^

Adjunctive medical management is patient specific, and few specific recommendations for missile emboli exist. Though no strong evidence suggests it as a necessity, prophylactic antibiotics should be considered. Intracardiac emboli may require post-injury bacterial endocarditis prophylaxis as no bullet or missile embolus is ever deemed sterile.^5^ If antibiotics are used, 48 hours of a first generation cephalosporin may be used in any high-velocity gunshot or shotgun injury, with addition of an aminoglycoside in the case of soft tissue cavitation which may house contaminated debris swept in upon bullet impact.^40^–^42^ Anticoagulation for pulmonary arterial and systemic venous emboli may be necessary in adult patients for 12 months if patients remain asymptomatic. Of note, lead toxicity does not need to be monitored with retained cardiac or vascular bullets, except when the bullet is exposed to joint synovial fluid or bone marrow.^43^ Serial imaging during hospital admission and outpatient follow up is required especially for patients who are chosen to be treated conservatively without surgical intervention. Finally, regardless of conservative or surgical management, counseling about complications the patient may experience should include seeking medical attention for fever, chest pain, palpitations, shortness of breath, or anxiety about retained cardiac missiles.^27^

## CONCLUSION

Management of intravascular and intracardiac missile emboli is not common knowledge. This review offers a synthesis of existing literature. It is our hope that this article provides a central source of reference, maximizing expedient decision making. It must be noted, however, that we only offer a model of an approach to missile emboli, and high level recommendations cannot yet exist given current understandings of missile emboli management. Intravascular and cardiac missile emboli have significant morbidity and mortality associated with them, and they are encountered frequently enough to merit significant attention by those who work with trauma patients. As represented in our case, there is also undeniable risk of complications associated with missile embolus removal, and it is suggested by our review that our patient may have done well without removal of the bullet. In the case of our patient, it was decided that a retained .38 caliber bullet in the right ventricle had substantial risk for further complication since it could not be reliably shown before open surgical intervention whether the bullet was freely moving within the right ventricle, so removal was deemed necessary. This was not without consequence as the patient required drainage of significant pleural and pericardial fluid collections before stabilizing postoperatively. Our patient’s surgical complications provide yet another example of why the decision to remove or retain missile emboli is such a point of controversy, and further emphasize the need for a thorough discussion of risks and benefits of interventions with the patient and available family. As more reports become available, recommendations on management will hopefully become more robust. Until then, the level of intervention must be made on a case-by-case basis by the clinicians and their informed patients.

## Figures and Tables

**Video:** Bullet echo 0.5x.
